# Monocyte-to-lymphocyte ratio as an inflammatory biomarker for predicting diabetic peripheral neuropathy in type 2 diabetes mellitus patients

**DOI:** 10.1186/s12902-025-02129-2

**Published:** 2025-12-26

**Authors:** Jie Li, Fan Hu, Xiaoqing Lu, Yaoyao Shen, Jinhua Chen, Hong Zhang

**Affiliations:** Department of Neurology, Jiangxi Provincial People’s Hospital, The First Affiliated Hospital of Nanchang Medical College, Nanchang, Jiangxi China

**Keywords:** Monocyte-to-lymphocyte ratio, Inflammation, Type 2 diabetes mellitus, Diabetic peripheral neuropathy

## Abstract

**Background:**

Type 2 diabetes mellitus (T2DM) is an intricate metabolic disorder often accompanied by low-grade inflammation. This study aimed to investigate the relationship between the monocyte-to-lymphocyte ratio (MLR) and diabetic peripheral neuropathy (DPN) in patients with T2DM.

**Method:**

A total of 236 individuals diagnosed with T2DM participated in the research. Clinical parameters were assessed, including the Toronto Clinical Neuropathy Score (TCNS), Compound Muscle Action Potential (CMAP), Sensory Nerve Action Potential (SNAP), nerve conduction velocity (NCV), complete blood count, biochemical markers, and inflammatory indicators. These parameters were analyzed and compared, followed by logistic regression and receiver operating characteristic (ROC) curve analyses.

**Results:**

The findings demonstrated that patients with DPN were generally older and had higher MLR levels and glycated hemoglobin levels, lower high-density lipoprotein cholesterol(HDL-C), longer disease duration, and higher FPG compared to patients without DPN. Additionally, CMAP and SNAP of median nerve (MN), ulnar nerve(UN), peroneal nerve (PN), and tibial nerve (TN) were significantly lower in the higher MLR group than in the higher MLR group( *P* < 0.05). ROC analysis indicated that MLR had an area under the curve (AUC) of 0.625, suggesting a limited discriminative ability to identify DPN.

**Conclusion:**

This study underscores the potential ability of MLR as a predictive biomarker for DPN in patients with T2DM, emphasizing the important role of inflammation in the development of this condition. But it has a low performance for DPN diagnosis.

**Clinical trial number:**

Not applicable.

## Introduction

It is known that T2DM is a multifaceted metabolic disorder. It results in persistent high blood sugar levels and considerable health issues worldwide due to insulin resistance and inadequate insulin secretion. The incidence of T2DM has surged to epidemic levels, impacting millions of individuals across the globe and posing significant public health challenges. This condition is closely associated with various complications, such as cardiovascular diseases, kidney damage, and nerve disorders [1]. Effective management strategies is essential for enhancing health outcomes. One notable complication is DPN, which is often overlooked in clinical settings. DPN is one of the most common yet frequently unrecognized complications of the disease which affects nearly 50% of individuals with diabetes [2]. The symptoms of DPN can significantly reduce the quality of life, but there is no recognized treatment to prevent the progression of DPN or directly repair damaged nerves. So early identification and screening play a crucial role in the management and treatment of DM.

Recent studies have revealed a strong link between inflammation and the development of diabetes-related complications. Inflammation is thought to play a crucial role in the onset of DPN. It showed that inflammatory markers could be useful for diagnosis or as targets for treatment. Symptom signs and electrophysiological examinations are the most important and commonly used methods in clinical assessment of DPN. NCS is the ‘gold standard’ for diagnosing large fiber neuropathies. It’s advantages include objectivity, high equipment availability, and ease of implementation. But it is unable to detect small fiber damage, which may result in the inability to detect neuropathy at an early stage in prediabetes. Peripheral blood tests offer a simpler and less invasive alternative to detect effective predictive indicators of DPN Compared with electrophysiological examination. Among these markers, the MLR has gained attention as a potential biomarker that reflects the body’s inflammatory state and may offer important insights into the progression of DPN [3]. The absolute count stability of MLR is good, MLR still exhibits good stability even when physiological, pathological, and physical factors affecting white blood cell count. However, existing researches were less investigating the relationship about MLR and DPN. It indicated that there’s a significant gap in our understanding of the underlying mechanisms and the potential clinical applications of MLR in this area.

The primary aim of our study is to investigate the MLR as a potential biomarker of DPN. This research seeks to enrich the current body of literature by identifying new biomarkers. It will be conducive to the early diagnosis and intervention of DPN. The results of this research may help create personalized clinical protocols that use the MLR as a dependable biomarker for tracking disease progression and informing treatment approaches in managing T2DM and its related neuropathies.

### Subjects

We conducted a retrospective analysis of 236 individuals diagnosed with T2DM participated in this study. All of patients were recruited from the inpatient department of Jiangxi Province People’s Hospital between January 1 and December 31, 2023. Clinical Data collection was carried out from all participants after obtaining informed consent and receiving approval from an ethical review board. The diagnostic criteria for T2DM followed the standards established by the American Diabetes Association [4]. DPN was diagnosed with the unified DPN screening protocol, which developed by the Chinese Diabetes Society of the Chinese Medical Association in 2010, based on the recommendations provided by the ADA in 2005 [5, 6] and the Toronto consensus [7]. Patients were classified into two categories: DPN (neuropathy symptoms plus at least one DSPN sign, or no symptoms but at least two DSPN signs, or abnormal NCS); no DPN (no neuropathy symptoms, no DSPN signs, and normal NCS, or neuropathy symptoms alone or one DSPN sign). The DM patients had no other relevant complications in addition to retinal vascular disease and peripheral neuropathy. All patient’s age ranges from 20 to 75 years.

Patients were excepted with a history of long-term or heavy alcohol consumption (i.e., an average daily intake of more than 250 g, sustained The duration of alcohol consumption was more than 10 years, and often without food ), occupational exposure to neurotoxins, peripheral nerve injury, signs of radicular irritation, cervical spondylosis, lumbar disc herniation, thyroid disease, and those taking painkillers for non-numbness pain relief were excluded. Additionally, individuals with vitamin B12 deficiency, leprosy, HIV positivity, pregnancy/lactation, organic bladder dysfunction, liver and kidney dysfunction, factors affecting NCV(such as mechanical pressure, freezing, muscle atrophy, chemical drugs), cardiovascular diseases (including stroke, myocardial infarction, atherosclerosis), and conditions that elevate inflammatory markers (like tumors or pneumonia) were not included. Informed consent was duly obtained from each participant following a comprehensive explanation of the study’s purpose and methodologies.

### Detection and definition of MLR

Venous blood was collected in the morning after an overnight fast after admission. Complete blood count was performed on the specimen using a Beckman Coulter DxH 800 instrument. MLR was is calculated by dividing the monocyte count by the lymphocyte count.

### Data collection

Data collection involved documenting a variety of parameters, such as the patients’ sex, age, medical history, duration of diabetes, and the presence of hypertension (HTN). body mass index (BMI), fasting plasma glucose (FPG), hemoglobin A1c (HbA1c), HDL-C, the TCNS, alanine aminotransferase (ALT), creatinine (Crea), total cholesterol(TC), fibrinogen(FIB), MLR, platelet-to-lymphocyte ratio(PLR), and neutrophil to lymphocyte ratio(NLR) The electrophysiology assessment included NCV, CMAP and SNAP. Along with various biochemical parameterswere carefully recorded.

### Electrophysiology measurement

Electrophysiology measurement were evaluated by two neurologists with an electromyography (EMG) machine (Keypoint 9033A07, Dantec Co). Peripheral nerve function is assessed via measuring SNAP, motor and sensory NCV and CMAP. To perform this assessment, nerve stimulation techniques were used on the motor and sensory nerve, including MN, UN, PN and TN in both limbs. Throughout the procedure, the local skin temperature was carefully maintained between 32 and 33 °C. Any variables that exceeded the mean ± 2 standard deviations, as established in our laboratory, were considered abnormal.

### Statistical analysis

For the purpose of statistical evaluation, we employed SPSS software version 20.0 to analyze the gathered data. The Kolmogorov-Smirnov test was used to examine the normality of distribution. Continuous variables that adhered to a normal distribution were subjected to analysis using the Student’s t-test for intergroup comparisons. Conversely, data that did not satisfy this normality assumption were expressed in terms of their medians. Categorical variables were expressed as counts and percentages, and we employed the χ2 test to compare these variables. To assess the impact of relevant indicators, we conducted binary logistic regression analysis on the significant factors, presenting the results as adjusted odds ratios (OR) along with their corresponding 95% confidence intervals (CI). Additionally, we constructed ROC curves for further evaluation. CMAP and SNAP in the different MLR groups were analyzed by the Student’s t-test. Statistical significance was established with a threshold of P values below 0.05.

## Results

### General data

Table 1 outlines the clinical characteristics of the study. In comparison to the non-DPN group, individuals in the DPN group showed significantly higher values in several areas: age (*P* < 0.001), duration of diabetes(*P* < 0.001), prevalence of HTN(*P* = 0.016), HbA1c levels(*P* = 0.001), TCNS (*P* < 0.001), PLR (*P* = 0.023), NLR(*P* = 0.020) and MLR(*P* = 0.007). On the other hand, BMI(*P* = 0.033) and HDL-C (*P* = 0.001) were significantly lower in the DPN group. However, there were no significant differences noted between the two groups regarding FPG, TC, ALT, Crea, and FIB.

**Table 1:** Characteristics of the study population

Variables	Total	DPN	Non-DPN	*P*-value
N(male/female)	236(138:98)	118(69:49)	118(70:48)	0.895
Age (years)	58.7 ± 12.3	61.9 ± 11.2	55.6 ± 12.5	0.000
Duration(years)	6.5 ± 6.4	8.6 ± 7.5	4.5 ± 4.3	0.001
HTN, n (%)	71(30.1)	44(37.3)	27(22.9)	0.016
BMI (kg/m2)	24.2 ± 3.9	23.7 ± 3.9	24.8 ± 3.8	0.033
TCNS	0.9 ± 1.5	1.4 ± 1.8	0.4 ± 0.8	0.000
HbA1c (%)	8.9 ± 2.3	9.4 ± 2.5	8.3 ± 2.0	0.001
FPG (mmol/L)	9.4 ± 4.2	9.8 ± 4.3	8.9 ± 4.0	0.09
ALT (U/L)	28.3 ± 23.1	27.0 ± 20.6	29.5 ± 25.4	0.41
Crea (µmol/L)	66.8 ± 23.5	69.9 ± 25.3	63.8 ± 21.2	0.49
TC (mmol/L)	4.7 ± 1.1	4.7 ± 1.1	4.7 ± 1.0	0.085
HDL-C (mmol/L)	1.0 ± 0.28	1.0 ± 0.3	1.1 ± 0.3	0.001
FIB	2.9 ± 0.7	3.0 ± 0.8	2.8 ± 0.7	0.123
MLR	0.28 (0.21,0.37)	0.28 (0.21,0.37)	0.23 (0.19,0.30)	0.001
PLR	111.29 (88.60,153.80)	115.27 (95.65,160.56)	105.35 (84.90,144.20)	0.023
NLR	1.87(1.41,2.62)	2.05 (1.54,2.70)	1.70(1.36,2.43)	0.020

The data are presented as means ± standard deviation (S.D.). For normally distributed data, values are shown as means ± S.D. and analyzed using Student’s t-test. In contrast, for data that do not follow a normal distribution, results are expressed as medians with interquartile ranges (IQR) and analyzed using nonparametric tests, specifically the Wilcoxon test. Categorical variables are represented as frequencies and proportions, with analyses performed using the chi-squared (χ²) test. Statistical significance is highlighted in bold (*P* < 0.05). The following abbreviations are used: ALT, alanine aminotransferase; BMI, body mass index; Crea, creatinine; FPG, fasting plasma glucose; HbA1c, hemoglobin A1c; HDL-C, High density lipoprotein cholesterol; FIB, fibrinogen; HTN, hypertension; NLR, neutrophil to lymphocyte ratio; TC, total cholesterol; TCNS, Toronto clinical neuropathy score; platelet-to-lymphocyte ratio, MLR, monocyte-to-lymphocyte ratio

### Binary logistic regression analysis

To explore the connection between MLR and DPN, variables were further subjected to multivariate regression analysis that showed statistical significance in univariate regression analysis. we conducted a binary logistic regression analysis using the enter method. We developed three models for this purpose. Model 1 focused solely on MLR, yielding a p-value of 0.004. In Model 2, we expanded the analysis by including age and duration along with the predictors from Model 1, which resulted in a p-value of 0.006. Finally, Model 3 further enhanced the predictors by adding BMI, HDL-C and HbA1c to those in Model 2, leading to a p-value of 0.029, as detailed in Table 2. Hosmer-Lemeshow test indicates good calibration performance for model 3 (Chi-square Value 9.8, *P* = 0.279).

**Table 2:** MLR associated with the presence of DPN in logistic regression (enter method)

		MLR	
	β(S.E)	OR (95% CI)	*P*-value
M1	2.642(0.909)	14.038(2.362–83.433)	0.004
M2	2.467(0.899)	11.783 (2.022–68.654)	0.006
M3	1.933(0.887)	6.91(1.214–39.329)	0.029

Data are presented as regression coefficient (standard error), odds ratio (95% CI) and P-value. Logistic regression analysis (enter method) was used to evaluate the association of MLR and DPN after adjusting other clinical and biochemical variables. Bold indicates statistical significance (*P* < 0.05). M1 is a regression model including just MLR; M2 adds age, duration to the predictors of M1; M3 adds BMI, HDL-C and HbA1c to the predictors of M2

### Sensitivity, specificity analysis and ROC

**Fig. 1:**
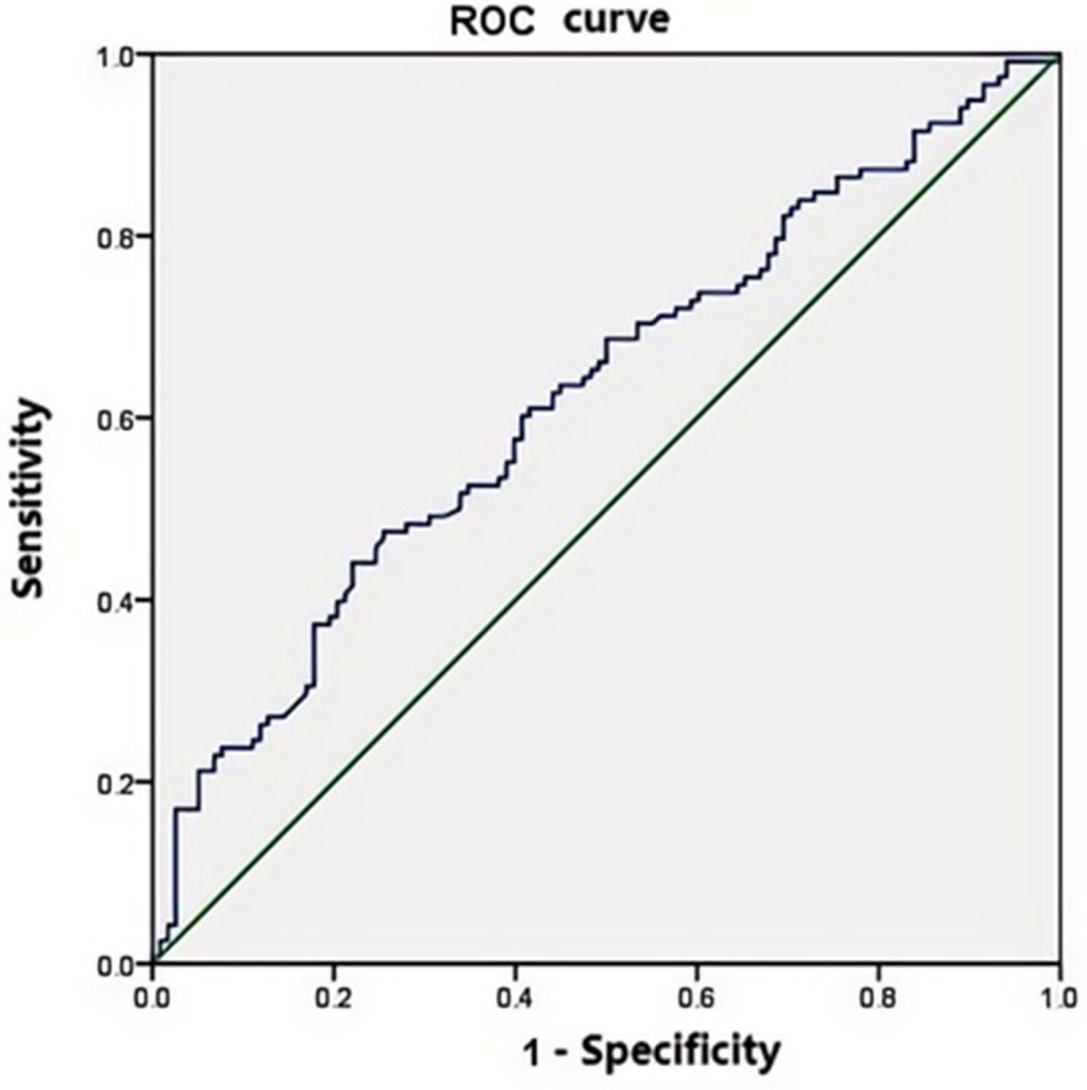
ROC curve of MLR for diagnosing DPN. AUC = 0.625 (*P* = 0.001). MLR, monocyte-to-lymphocyte ratio ratio; DPN, diabetic peripheral neuropathy; AUC, area under ROC curve

The impact of MLR on diagnosing DPN was assessed using ROC curve, as illustrated in Fig. 1. The area under the ROC curve was found to be 0.625(*P* = 0.001). An optimal cut-off value for MLR was determined to be 0.29, which resulted in a sensitivity of 47.5% and specificity of 74.6%.The positive predictive value of the ROC curve is 65.2%, and the negative predictive value is 58.7%.

### CMAP and SNAP in the different MLR groups

**Fig. 2:**
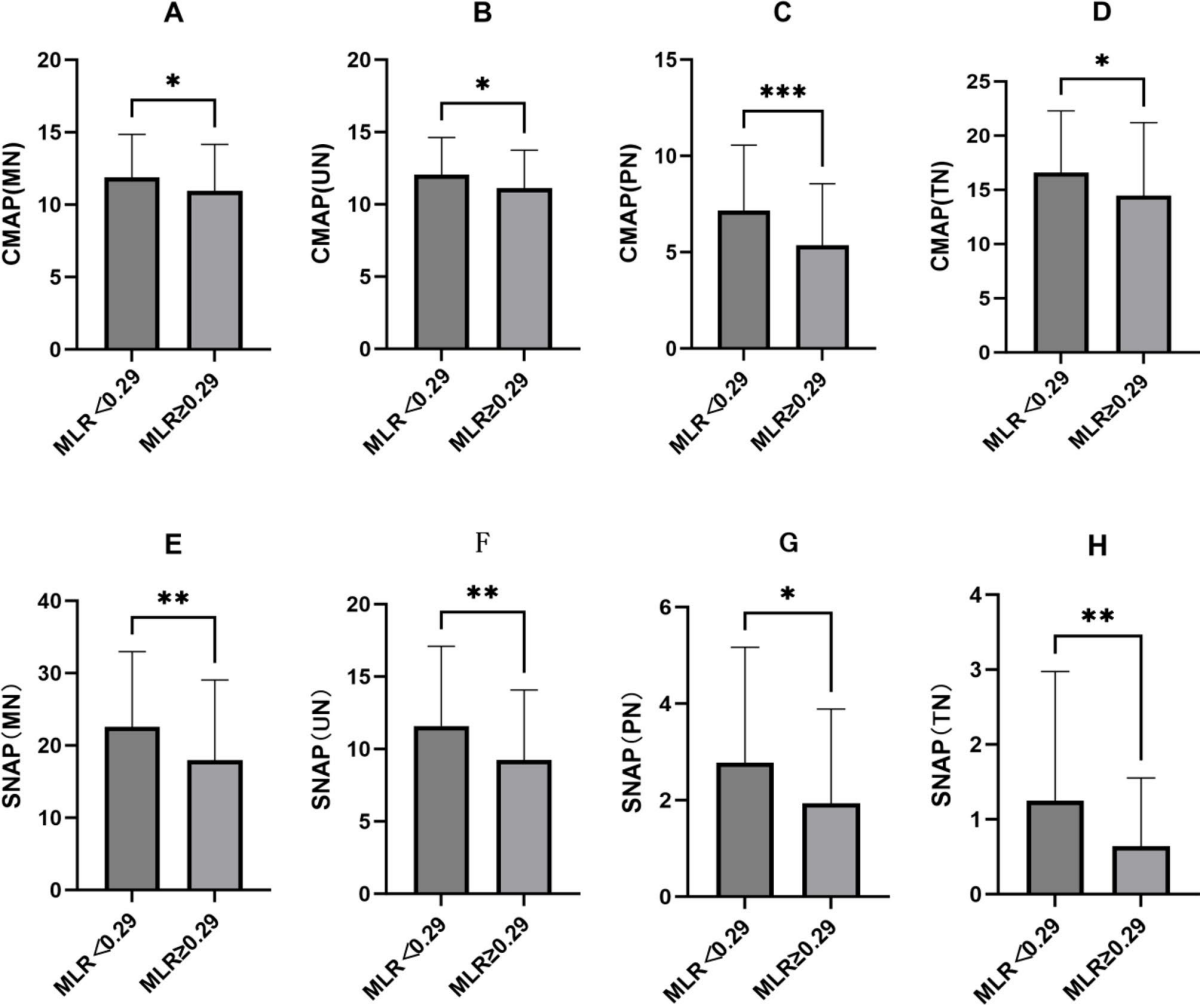
CMAP and SNAP in the different MLR groups were analyzed by the Student’s t-test. **P < 0.05, **P < 0.01, ***P < 0.001. MN, mediannerve; UN, ulnar nerve; PN, peroneal nerve; TN, tibial nerve; MLR, monocyte-to-lymphocyte ratio ratio; CMAP, Compound Muscle Action Potential SNAP, Sensory Nerve Action Potential

MLR was significantly highest in patients with DPN (Table 1, *P* = 0.001). Patients were divided into two groups based on PFR (the lower group < 0.29, the higher group ≥ 0.29). We analyzed the differences between CMAP and SNAP among the two groups. CMAP and SNAP of MN, UN, PN and TN were significantly lower in the higher MLR group than in the higher MLR group(Fig. 2A to D, *P* < 0.01–0.001; E to H, *P* < 0.05–0.01).

## Discussion

The pathogenic mechanisms of DPN include increased oxidative/nitrosative stress, activation of the polyol and protein kinase C pathways, activation of polyADP ribosylation, and activation of genes involved in neuronal damage, cyclooxygenase-2 activation, endothelial dysfunction, altered Na+ /K+ -ATPase pump function, impaired C-peptide-related signaling pathways, endoplasmic reticulum stress, and low-grade inflammation. Glucose, lipoproteins, oxidized and glycated proteins bind to neuronal receptors. These receptors can trigger inflammatory signaling [8]. Additionally, chronic hyperglycemia fosters the formation of advanced glycation end products (AGEs), it give rise to inflammatory responses and further oxidative stress When AGEs bind to their receptors in neurons and Schwann cells [9]. Our study showed that patients with DPN had significantly higher glycated hemoglobin levels and lower HDL-C, longer disease duration, and obvious FPG compared with patients without DPN. This suggests that longer disease duration and poorer glycemic control may be linked to inflammation, which may promote the development of DPN.

There’s increasing evidence of a contribution of low-grade inflammation in the development of DPN [10, 11]. Some researches demonstrated that inflammation was closed with diabetic complications, especially neuropathy. It suggested that inflammatory markers could be utilized as valuable diagnostic and prognostic tools in clinical settings [3, 8, 9, 12]. White blood cells and their subtypes are key indicators of inflammation. The NLR, PLR and MLR serve as potential biomarkers for various diseases and inflammatory conditions, including tumors, cardiovascular diseases, and other disorders [13, 14]. At present, there is limited research on the relationship between MLR and DPN. Our study investigated the predictive relationship between MLR and DPN, which is a relatively novel area of inquiry. To explore potential biomarkers for the early diagnosis of DPN, our study explored the connection between the MLR and DPN in a group of 236 patients diagnosed with T2DM. Our results indicated that patients suffering from DPN had notably higher levels of MLR, the higher MLR exhibited lower CMAP and SNAP. It revealed a possible association between inflammatory processes and the DM with neuropathic complications.

PLR, MLR, and NLR are a potential biomarker reflecting inflammation and immune response, and MLR has been confirmed as an important potential biomarker for various inflammatory diseases [15, 16].Recent studies have shown that elevated levels of MLR are closely associated with the occurrence and development of diabetes complications, including diabetic neuropathy (DN) and diabetic retinopathy (DR) [17]. Furthermore, MLR has been extensively researched in inflammation-related conditions, including cancer and cardiovascular diseases, and has proven to be a reliable biomarker of systemic inflammation [18, 19]. Therefore, a thorough analysis of the relationship between MLR and DPN may offer some insights into the pathogenesis and progression of this severe diabetic complication. As with Li, Z’s study [20], our results also demonstrated that patients with DPN exhibited a significantly higher MLR compared to those without DPN. DM patients suffer from microvascular lesions, leading to ischemic necrosis of nerve fiber axons particularly sensory nerve [21, 22]. Studies have shown that inflammation can cause vascular swelling and constriction, reducing blood flow. This results in increased ischemia and hypoxia in nerve cells, further damaging Schwann cells and contributing to neuropathy [9]. During an EMG test, the initial reduction in conduction amplitude and sensory NCV were observed early in DPN [23]. We explored the correlationship between MLR and the nerve conduction amplitudes of DPN patients. After adjusting for age, blood glucose fluctuations, and disease duration, our regression analysis found that MLR remains an independent risk factor for DPN. ROC curve evaluation showed that an MLR ≥ 0.29 suggests the possibility of DPN. We further analyzed CMAP and SNAP between the low MLR group and the high MLR group based on the cutoff value, and found that CMAP and SNAP were significantly lower in the higher MLR group. Our study sought to clarify the predictive significance of MLR as a biomarker for DPN, but its predictive ability was limited.

The mechanisms between MLR and DPN are still unclear. Monocytes play a crucial role in the non-specific immune system. Not only they phagocytose pathogens and cellular debris but also initiate immune responses by connecting the specific immune system through antigen presentation. Cytokines are primarily secreted by immune cells like T cells, macrophages, and neutrophils, though other cell types—including Schwann cells and glial cells in the central nervous system—also produce them. Pro-inflammatory cytokines play a crucial role in the development of DPN such as TNF-α, C-reactive protein, IL-1, IL-6, IL-8 and monocyte chemoat tractant protein-1 (MCP-1) [9, 24]. The mechanism by which MLR exerts its effect may be as follows: First, elevated MLR leads to changes in the levels of pro-inflammatory chemokines such as C-reactive protein, IL-1, IL-6, TNF-α, and MCP-1. These chemokines are key in recruiting and activating monocytes and leukocytes, driving subsequent inflammatory responses in patients with DPN. the DPN group exhibits elevated MLR, which suggests a potential link to persistent monocyte activation and inhibited lymphocyte function in a chronic inflammatory state. MLR is an indicator that comprehensively reflects enhanced inflammation and immune imbalance. Elevated MLR levels is associated with upregulation of pro-inflammatory factors and activation of monocyte-macrophages. The results of this study provide new evidence supporting MLR as a potential predictive biomarker for DPN.

The limitations of this study require careful attention. First, the relatively small sample size may limit how widely our findings can be applied, as a larger group could yield more reliable data on the relationship between MLR and DPN. It is necessary to conduct longitudinal studies owing to lacking the ability to establish cause-and-effect relationships of the cross-sectional study. Future research should focus on including larger, multi-center groups and investigating the long-term effects of MLR on the progression of DPN.

In conclusion, the MLR was significantly increased in patients with DPN. Although the MLR is an independent risk factor for DPN in Chinese patients with T2DM, its predictive was limited for DPN diagnosis.It suggested that inflammation may significantly contributed to the development of diabetic neuropathy. These findings highlight the necessity of incorporating inflammatory markers into clinical evaluations, which could aid in the early identification of DPN. We should aim to confirm these findings and investigate therapeutic strategies that target inflammatory pathways to improve clinical outcomes in the future.

## Data Availability

The data that support the findings of this study are available from the corresponding author upon reasonable request. The data are not publicly available due to privacy or ethical restrictions.
